# Towards Personalized Medicine in Melanoma: Implementation of a Clinical Next-Generation Sequencing Panel

**DOI:** 10.1038/s41598-017-00606-w

**Published:** 2017-03-29

**Authors:** Blanca de Unamuno Bustos, Rosa Murria Estal, Gema Pérez Simó, Inmaculada de Juan Jimenez, Begoña Escutia Muñoz, Mercedes Rodríguez Serna, Victor Alegre de Miquel, Margarita Llavador Ros, Rosa Ballester Sánchez, Eduardo Nagore Enguídanos, Sarai Palanca Suela, Rafael Botella Estrada

**Affiliations:** 1 Department of Dermatology, Hospital Universitari i Politecnic La Fe, Valencia, Spain; 2 Molecular Biology Laboratory, Service of Clinical Analysis, Hospital Universitari i Politecnic La Fe, Valencia, Spain; 3 Department of Dermatology, Hospital General Universitario de Valencia, Valencia, Spain; 4 Department of Pathology, Hospital Universitari i Politecnic La Fe, Valencia, Spain; 5 Department of Dermatology, La Plana Hospital, Villarreal, Castellón Spain; 6 Department of Dermatology, Department of Dermatology, Instituto Valenciano de Oncología, Valencia, Spain

## Abstract

Molecular diagnostics are increasingly performed routinely in the diagnosis and management of patients with melanoma due to the development of novel therapies that target specific genetic mutations. The development of next-generation sequencing (NGS) technologies has enabled to sequence multiple cancer-driving genes in a single assay, with improved sensitivity in mutation detection. The main objective of this study was the design and implementation of a melanoma-specific sequencing panel, and the identification of the spectrum of somatic mutations in a series of primary melanoma samples. A custom panel was designed to cover the coding regions of 35 melanoma-related genes. Panel average coverage was 2,575.5 reads per amplicon, with 92,8% of targeted bases covered ≥500×. Deep coverage enabled sensitive discovery of mutations in as low as 0.5% mutant allele frequency. Eighty-five percent (85/100) of the melanomas had at least one somatic mutation. The most prevalent mutated genes were *BRAF* (50%;50/199), *NRAS* (15%;15/100), *PREX2* (14%;14/100), *GRIN2A* (13%;13/100), and *ERBB4* (12%;12/100). Turn-around-time and costs for NGS-based analysis was reduced in comparison to conventional molecular approaches. The results of this study demonstrate the cost-effectiveness and feasibility of a custom-designed targeted NGS panel, and suggest the implementation of targeted NGS into daily routine practice.

## Introduction

Malignant melanoma is the most aggressive form of skin cancer, with a poor prognosis for patients with metastatic disease. Melanomas are currently classified based on clinical and histologic characteristics of the primary tumors; in addition, it has been described that distinct patterns of genetic alterations contribute to the development of the different subtypes of primary melanoma. It is well known that superficial spreading melanoma (SSM) and nodular melanoma (NM) are associated with *BRAF* or *NRAS* mutations; acral lentiginous melanoma (ALM), lentigo maligna (LM), and mucosal melanoma are more often associated with *KIT* mutations; and ocular melanomas are not associated with these common oncogenes, but rather with *GNAQ* or *GNA11* alterations^1, 2^. A recent integrative analysis of cutaneous melanoma from The Cancer Genome Atlas (TCGA) has established a new genomic classification into four subtypes, based on the identification of the most prevalent mutated genes [BRAF, RAS, NF1 and triple-wild type (wt) subtypes]^3^.

The advances on melanoma molecular pathogenesis have opened a new insight for the management of advanced melanoma due to the development of novel therapies that target causative genetic events, and improve disease free survival and overall survival^4^. The selective BRAF kinase inhibitors (Vemurafenib and dabrafenib) are effective in *BRAF* mutant melanoma; MEK inhibitors (trametinib and cobimetinib) show efficacy against both *BRAF*- and *KRAS/NRAS*-driven tumors; KIT inhibitors (imatinib, dasatinib, sunitinib and nilotinib) have demonstrated clinical responses in melanoma arising from acral, mucosal, and chronic sun-damaged cutaneous sites; and additionally, there are novel therapeutic monoclonal antibodies targeted against immunosuppressive molecules such as CTLA4, PD-1 and PD-L1.

Therefore, molecular diagnostics are increasingly performed routinely in the diagnosis and management of patients with melanoma. Conventional molecular analyses for detecting cancer somatic alterations have relied on methods such as Sanger sequencing and real-time quantitative polymerase chain reaction (RQ-PCR). These approaches have the limitation that are performed separately for each gene and therefore consume a high turn-around time. Moreover, Sanger sequencing has a relatively low sensitivity, and sometimes it can be challenging to detect somatic mutations, especially when tumor material is mixed with normal tissue. In this context, the development of next-generation sequencing (NGS) technologies has enabled to massively analyze millions of DNA segments in parallel, thus allowing to sequence multiple cancer-driving genes in a single assay, with improved sensitivity in mutation detection. One of the developed NGS methodologies is the new Ion Torrent sequencing platform, based on the detection of hydrogen ions released on each cycle of DNA polymerization. It has been described to be cost and time effective^5^, and its applicability in formalin-fixed and paraffin-embedded (FFPE) specimens with small amounts of DNA has been proved in several reports^6, 7^. The use of a multi-gene screening panel may potentially allow a more personalized approach to cancer therapy by identifying less common but potentially actionable mutations.

In the present study, we have used Ion Torrent sequencing technology with the Personal Genome Machine (PGM) and a custom AmpliSeq Panel including 35 genes. The main objective was the design and implementation of a melanoma-specific sequencing panel, based on the inclusion of relevant melanoma-genes with clinical diagnostic, prognostic or treatment value. Moreover, we aimed to describe the mutation profile in a series of primary melanoma samples in order to provide new insights into the molecular subclassification of melanoma. The translation of these study results may provide further understanding of the molecular alterations that lead to the development of melanoma, and therefore may contribute to the improvement of a personalized medicine.

## Results

### Next-generation sequencing quality metrics and sensitivity assay

**Figure 1:**
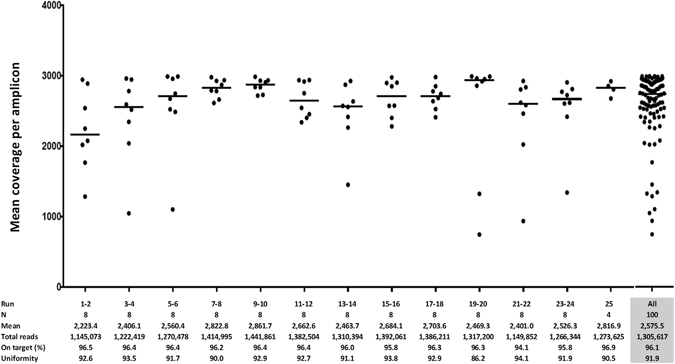
Quality metrics for 25 runs including mean coverage, total reads, reads on target and uniformity.

Quality control analysis revealed excellent performance of the panel (Fig. 1). Of note, the average total reads per sample was 1,305,617 with an average coverage of 2,575.5 reads per amplicon (Figure S1). Moreover, 97.8% of targeted bases covered ≥100x and 92.8% ≥500x. The coverage uniformity of amplicon sequencing was very high with an average of 91.9%. In addition, the sensitivity assay performed with serial dilutions in two independent experiments confirmed a high sensibility of the IonTorrent PGM (Table 1). *BRAF* mutations could be detected at an allele frequency as low as 0.05% with a rate of false reads of 0.1%. Therefore adjusting the sensitivity to our error-rate, the limit of detection was 0.5% (Figure S2).

**Table 1:** Sequencing results of serially diluted DNA isolated from two adenocarcinoma cell lines (HT-29 y Caco-2) with known variants in *BRAF* and *MC1R* genes.

HT-29 in Caco-2 (variant frequency)	*BRAF* gene, p.V600E (heterozygous)				*MC1R* gene, p.R160W (heterozygous)			
	Allele frequency (%)		Coverage (x)		Allele frequency (%)		Coverage (x)	
	Exp 1	Exp 2	Exp 1	Exp 2	Exp 1	Exp 2	Exp 1	Exp 2
Undiluted (50%)	57.12	52.85	2027	2182	61.42	64.48	1894	2329
1:1 (25%)	26.22	25.69	4366	5780	32.66	32.93	2103	2708
1:3 (12.5%)	13.13	12.85	5620	3507	14.84	15.53	2182	2524
1:24 (2%)	1.82	1.75	3720	3523	1.95	1.21	1894	1981
1:49 (1%)	1.12	0.68	4266	3927	0.80	0.87	2967	2631
1:99 (0.5%)	0.65	0.64	3977	3430	0.57	0.34	2624	1458
1:999 (0.05%)	0.04	0.10	4993	2945	0.03	0.19	3065	3138

Exp: Experiment.

### Mutations detected by Next-generation sequencing in melanoma

#### Mutation prevalence

Sequencing identified on average 56.6 total variants and 8.9 exonic variants per sample, that were subsequently filtered in order to exclude variants without impact on protein function. After filtering, a total of 135 different pathogenic variants were finally reported in all the samples (Table S1). In the entire cohort, 94% (94/100) had at least one pathogenic variant, and 51% (51/100) had ≥3. Without considering *MC1R* polymorphisms, 85% (85/100) of the melanomas had at least one somatic mutation.

**Figure 2:**
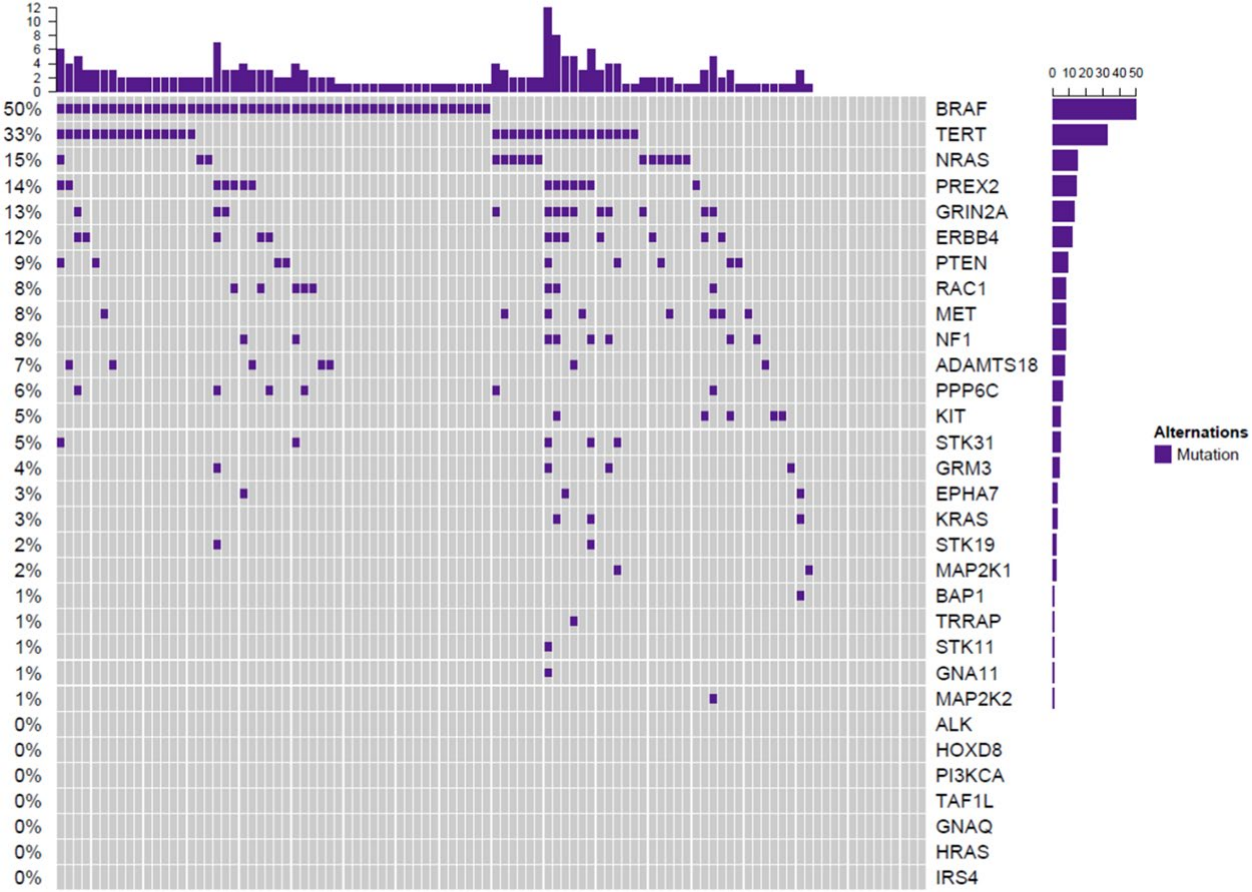
Frequency of somatic gene mutations. Each column represents 1 sample and each row represents 1 gene. The column on the left indicates the percentage of samples with specific gene mutation.

The most prevalent mutated genes were *BRAF* (50%; 50/100), *NRAS* (15%; 15/100), *PREX2* (14%; 14/100), *GRIN2A* (13%; 13/100), and *ERBB4* (12%; 12/100). All the mutated genes are represented in Fig. 2. Regarding *BRAF* gene, the most frequent mutation was p.V600E (80%; 40/50), followed by p.V600K (10%; 5/50), p.K601E (4%; 2/50), p.L597R (2%; 1/50), p.L584F (2%; 1/50), and p.G464R (2%; 1/50). The most frequent affected codon in *NRAS* was Q61 (87,6%; 14/16), followed by G12 (6,3%; 1/16) y E62 (6,3%; 1/16). *KRAS* oncogene mutations were identified in 3% (3/100), and the most prevalent mutation was p.Q61R (75% ; 2/3); *KIT* mutations were detected in 5% (5/100) of the samples, and two of them showed the hotspot mutation p.L576P. In regard to *MAP2K1* and *MAP2K2*, the overall prevalence was 3% (3/100). *PTEN* mutations were present in 9% (9/100) of the melanomas, with a recurrent point mutation in two of the samples (p.F278L). *PIK3CA* mutations were not identified in our cohort. *NF1* mutations were detected in 8% (8/100) of the melanomas; and nonsense mutations were the most prevalent alteration [62.5% (5/8)], including one recurrent mutation present in two samples (p.R1362*). The overall prevalence of variations in *PREX2* and *GRIN2A* genes, was 28% (28/100) and 21% (21/100), with a total of 31 and 27 different variants dispersed throughout the entire gene, respectively. After filtering out, the prevalence of pathogenic variants was 14% (14/100) for *PREX2* gene and 13% (13/100) for *GRIN2A* gene. *ERBB4* variants were also identified in 15% (15/100) of the samples, and 80% (12/15) of them were predicted as pathogenic. Hotspot mutations were detected in *RAC1* (p.P29S and p.P69L) and *PPP6C* (p.D193Y, p.P209L, and p.R264C) in 8% and 6% of the samples, respectively. None of the melanomas harbored the hotspot activating mutation p.S722F in *TRRAP*, although we identified one sample with another *TRRAP* pathogenic variant (p.P814S).

Although paired tumor-normal samples were not collected, melanoma related polymorphisms were analyzed. *MC1R* polymorphisms were detected in 66% (66/100) of the patients; 4% (4/100) showed polymorphisms in *CDK4*; and 2% (2/100) in *MITF*. The variant p.V60L in *MC1R* was the most prevalent polymorphism [36% (36/100)], followed by p.V92M [17% (17/100)] and p.D294H [9% (9/100)]. Regarding *CDK4* gene, two of the samples showed the polymorphism p.R24H, one p.R24C and one p.L22R. Moreover, the *MITF* p.E318K variant was detected in two additional samples.

#### Associations between mutations and clinicopathological characteristics

The prevalence of gene mutations varied by melanoma location, as most of the *KIT* mutated melanomas were distributed in chronically sun-damaged skin and acral melanomas, and were significantly associated with ALM [p = 0.004; OR = 4.4 (1.6–12.1)]. Conversely, *BRAF* mutations were associated with histological subtypes other than ALM [p = 0.013; OR = 0.5 (0.2–0.8)]. In adition, *GRIN2A* and *RAC1* mutations were also associated with chronically sun-exposed melanomas [p = 0.011; OR = 5.9 (1.5–23.2) and p = 0.026; OR = 3.2 (1.1–9.2), respectively]. Interestingly, *NF1* mutations were more prevalent in melanomas with increased Breslow thickness [p = 0.025; OR = 2.16 (1.10–4.26)], ulceration [p = 0.003; OR = 26.89 (3.11–232.10)], and fast-growing melanomas^8^ [p = 0.004; OR = 12.16 (2.26–65.37)]. Furthermore, *PREX2* and *GRIN2A* mutations were significantly more prevalent in melanomas with mitosis [p = 0.042; OR = 4.0 (1.0–15.4) and p = 0.023; OR = 6.2 (1.3–29.5), respectively]. All significant associations with clinicopathological characteristics are shown in Table 2. No correlation between mutations and clinical outcome was found.

**Table 2:** Associations of mutations with clinicopathological characteristics.

Variables	Mutational status		Total N	OR	95% CI	P-value
	Wildtype N (%)	Mutated N (%)				
***BRAF***	**50 (50%)**	**50 (50%)**				
**Histological subtype**						
LMM	5 (55.6)	4 (44.4)	9	0.5	0.2–0.8	0.013
SSM	29 (41.2)	40 (54.8)	69			
NM	8 (57.1)	6 (42.9)	14			
ALM	8 (100)	0 (0)	8			
***KIT***	**95 (95%)**	**5 (5%)**				
**Histological subtype**						
LMM	9 (100)	0 (0)	9			
SSM	68 (98.5)	1 (1.5)	69	4.4	1.6–12.1	0.004
NM	13 (92.9)	1 (7.1)	14			
ALM	5 (62.5)	3 (37.5)	8			
**Ulceration**						
No	73 (98.6)	1 (1.4)	74	13.3	1.4–124.9	0.024
Yes	22 (84.6)	4 (15.4)	26			
**Tumor stage**						
I–II	79 (97.5)	2 (2.5)	81	7.4	1.1–47.9	0.036
III–IV	16 (84.2)	3 (15.8)	19			
***PREX2***	**86 (86%)**	**14 (14%)**				
**Mitosis**						
<1	45 (93.8)	3 (6.3)	48	4.0	1.0–15.4	0.042
≥1	41 (78.8)	11 (21.2)	52			
***GRIN2A***	**87 (87%)**	**13 (13%)**				
**Sun related site**						
Non exposed	20 (95.2)	1 (4.8)	21			
Ocasionally exposed	56 (88.9)	7 (11.1)	63	3.2	1.1–9.2	0.026
Usually exposed	11 (68.8)	5 (31.3)	16			
**Mitosis**						
<1	46 (95.8)	2 (4.2)	48	6.2	1.3–29.5	0.023
≥1	41 (78.8)	11 (21.2)	52			
***RAC1***	**92 (92%)**	**8 (8%)**				
**Sun related site**						
Non exposed	21 (100)	0 (0)	21			
Ocasionally exposed	59 (93.7)	4 (6.3)	63	5.9	1.5–23.2	0.011
Usually exposed	12 (75)	4 (25)	16			
***NF1***	**92 (92%)**	**8 (8%)**				
**Breslow**						
≤1	45 (95.7)	2 (4.3)	47			
1–2	21 (100)	0 (0)	21	2.2	1.1–4.3	0.025
2–4	15 (78.9)	4 (21.1)	19			
>4	10 (76.9)	3 (23.1)	13			
**Ulceration**						
No	72 (97.3)	2 (2.7)	74	26.9	3.1–232.1	0.003
**Yes**	19 (73.1)	7 (26.9)	26			
**Growth rate**						
SGM	74 (97.3)	2 (2.7)	76	12.2	2.3–65.4	0.004
FGM	17 (70.8)	7 (29.2)	19			

LMM: Lentigo maligna melanoma; SSM: Superficial spreading melanoma; NM: Nodular melanoma; ALM: Acral lentiginous melanoma; SGM: Slow growing melanoma; FGM: Fast growing melanoma.

#### Concurrent molecular alterations in melanomas

**Figure 3:**
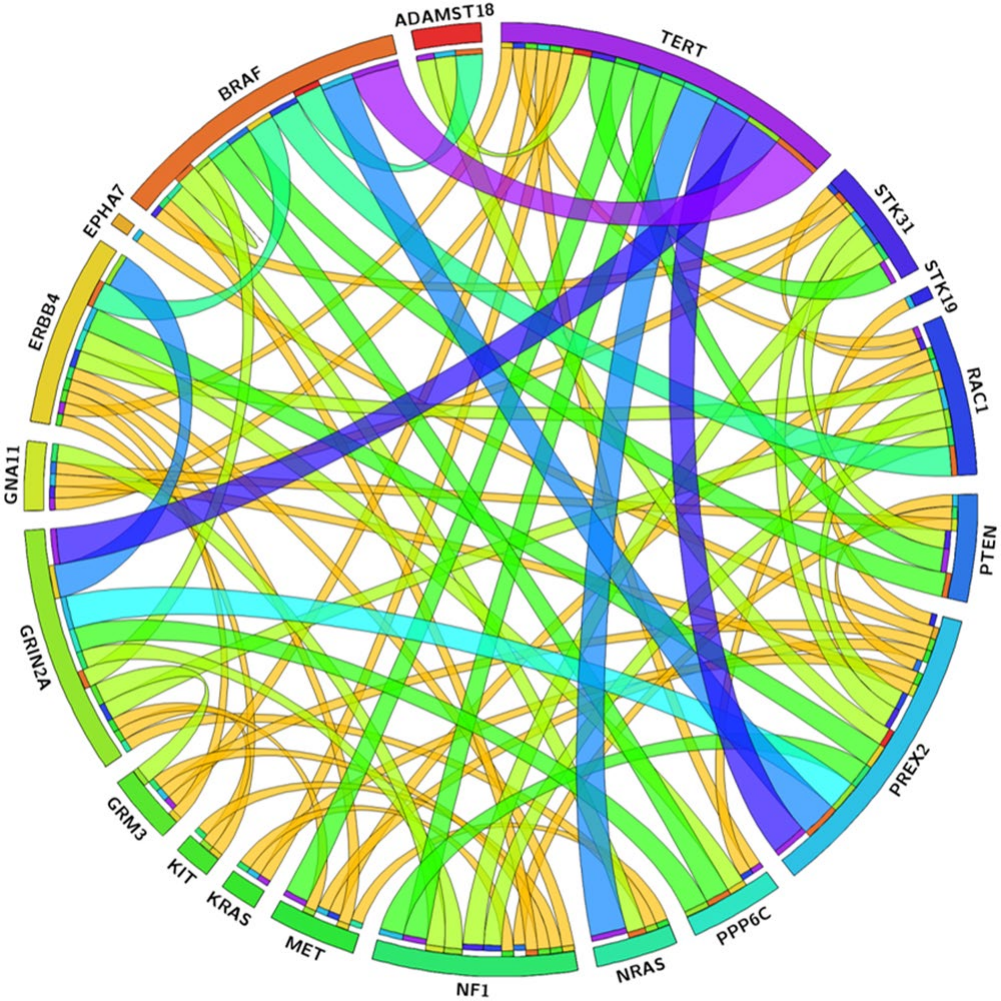
Circos diagram. Associations between the more prevalent genes.

Several genes harbored concurrent mutations among the studied genes (Fig. 3).We observed mutual exclusivity between *BRAF* hotspot mutations (p.V600E, p.V600K and p.K601E) and *NRAS/KRAS* mutations in almost all samples; however, one sample concurrently harbored *BRAF* p.V600E and *NRAS* p.Q61R mutations. Moreover, we found concurrent mutations in *BRAF* non-hotspot mutations (p.L584F and p.L597R) with *NRAS* (p.Q61L and p.G12S). Strikingly, one sample showed two concurrent *NRAS* mutations (p.Q61R and p.E62Q), another sample showed concurrent mutations on *KRAS* (p.Q61R and p.A146V), and another one two concurrent mutations in *KIT* (p.Y553S and p.Y578C). The clinicopathological features of patients with concurrent mutations are listed in Table 3.

**Table 3:** Clinicopathological characteristics of the samples with concurrent mutations in the four genomic subtypes.

No.	Gender	Age	Breslow (mm)	Anatomic site	Sun related site	Histological subtype	Gene	Amino acid change	Allele frequency (%)
**2**	M	79	2.7	Trunk	Occasional exposed	SSM	*BRAF*	p.Gly464Arg	6.8
							*NF1*	p.Arg711Cys	4.7
**28**	F	58	0.7	Lower extremities	Occasional exposed	SSM	*NRAS*	p.Glu62Gln	8.0
							*NRAS*	p.Gln61Arg	8.0
**58**	M	69	0.4	Trunk	Occasional exposed	SSM	*BRAF*	p.Leu597Arg	4.1
							*NRAS*	p.Gly12Ser	5.0
**62**	M	66	4.2	Head and neck	Usually exposed	LMM	*KRAS*	p.Gly12Val	80.8
							*NF1*	p.Trp1512*	30.8
**68**	M	54	0.7	Head and neck	Usually exposed	SSM	*BRAF*	p.Val600Glu	25.4
							*NF1*	p.Lys1844Th	6.3
**71**	F	85	3	Acral	Non exposed	ALM	*KIT*	p.Tyr553Ser	31.8
							*KIT*	p.Tyr578Cys	43.9
**80**	F	73	0.3	Upper extremities	Occasional exposed	SSM	*BRAF*	p.Val600Glu	2.0
							*NRAS*	p.Gln61Arg	3.0
**84**	F	57	2	Upper extremities	Occasional exposed	SSM	*BRAF*	p.Leu584Phe	23.1
							*NRAS*	p.Gln61Leu	24.0
**107**	M	59	6	Head and neck	Usually exposed	NM	*KRAS*	p.Gln61Arg	5.3
							*KRAS*	p.Ala146Val	10.6
							*NF1*	p.Gln1070*	13.5

M: Male; F: Female; SSM: Superficial spreading melanoma; LM: Lentigo Maligna; NM: Nodular melanoma; ALM: Acral lentiginous melanoma.

To verify the variant calling accuracy, *BRAF, NRAS*, *KRAS* and *KIT* mutated samples were subsequently analyzed by HRM, Sanger and RQ-PCR. *BRAF* mutations were detected by HRM and Sanger in 54% (27/50) of the melanomas; the average allele frequency of those wt samples was 4.4% with an average variant coverage of 229.5 reads. *NRAS* and *KRAS* mutations were confirmed by RQ-PCR in all the samples, and *KIT* mutations were confirmed in 80% (4/5) of the samples. Above mentioned concurrent mutations of *NRAS*, *KRAS* and *KIT* in the same sample were also confirmed by direct sequencing.

Ninety percent of the samples were correctly classified in four genomic subtypes^3^ (45 BRAF, 10 RAS, 4 NF1 and 31 triple-wt); however apart from the already mentioned, concurrent mutations were also identified between *NF1* and *BRAF*, and also between *NF1* and *KRAS*. Considering BRAF, RAS and NF1 subtypes together, a total of 31% (31/100) of our samples were triple-wt, and 9% (3/31) and 6% (2/31) of them harbored *KIT* and *PTEN* mutations, respectively (Figure S3).

### *TERT* promoter mutations

The prevalence of *TERT* promoter mutations was 33% (33/100), The most frequent somatic changes were −146C > T and −124C > T that were detected in 45% (15/33) and 30% (10/33) of the samples, respectively. Other recurrent changes identified were −124/−125CC > TT (6%; 2/33), −138/−139 CC > TT (15%; 5/33) and −57 A > C (3%; 1/33). We found mutual exclusivity between all the variants. *TERT* promoter mutations were observed in 32% (16/50) of *BRAF*, 47% (7/15) of *RAS* and 50% (4/8) of *NF1* subtypes. Mutations were significantly associated with female gender [p = 0.021; OR = 2.76 (1.16–6.54)], increased Breslow thickness [p = 0.001; OR = 2.07 (1.37–3.13)], ulcerated melanomas [p = 0.010; OR = 3.37 (1.33–8.56)], presence of mitosis [p = 0.001; OR = 4.63 (1.82–11.78)], absence of regression [p = 0.039; OR = 0.45 (0.21–0.96)], and fast-growing melanomas [p = 0.015; OR = 3.25 (1.25–8.42)]. Results of the univariate analysis are represented in Figure S4. In the multivariate analysis female gender [p = 0.001; OR = 6.50 (2.06–20.49)], increased Breslow thickness [p = 0.002; OR = 2.50 (1.40–4.50)], nodular histological subtype [p = 0,043; OR = 0,45 (0.21–0.97)], and the presence of mitosis [p = 0.014; OR = 4.48 (1.35–14.78)] were independently associated with *TERT* promoter mutations.

### Turn-around time and cost comparison

In order to compare NGS with conventional molecular testing under routine laboratory conditions, we calculated the turn-around time and costs for three commonly melanoma-related genes (*BRAF*, *NRAS*, *KIT*) in eight samples. Starting from FFPE samples, we were able to isolate DNA, prepare libraries and sequence eight samples within two sequencing runs on 318v2 chips in approximately three working days. The costs of consumables and laboratory personal for NGS was 415.5€ per sample. Grouping turn-around time analysis for *BRAF* (exon 15), *NRAS* (exons 2, 3 and 4) and *KIT* (exons 9, 11, 13 and 17) genes, the conventional methods resulted in approximately five working days. As expected, the mean turn-around time for NGS-based analysis was lower in comparison to routine methods. The total cost for conventional methods was more expensive in comparison to NGS (Table 4).

**Table 4:** Turn-around time and cost comparison between Next-generation sequencing and conventional molecular analysis.

Analysis		System	Time duration (min)	Costs** (€)
NGS analysis (8 samples; 35 genes)				
Quantification and sample dilution		Qubit	60	4
Library preparation		Veriti Thermal Cycler	310	1819.8
Emulsion PCR		One Touch	315*	238.8
Enrichment		One Touch ES	45	47.2
Sequencing (two 318 chip v2)		PGM System	360 (x2)	1214
Data processing and analysis		Ion Reporter	110	0
Laboratory personal costs			NA	288
Total			**1,560 (=26** **h)**	**3323.8**
Working days			**3**	**NA**
Analysis		Method		
Conventional molecular analysis (8 samples; 3 genes)				
Quantification		Nanodrop	30	0
*BRAF*	Exon 15	RQ-PCR HRM+ SS	510 (=8^1/2^ h)	460.2
*NRAS*	Exon 2/3/4	RQ-PCR (hibridization probes)+ SS	420 (=7 h)	1,225.6
*KIT*	Exon 9/11/13/17	Conventional PCR + SS	1,230 (20^1/2^ h)	1,555.4
Laboratory personal costs			NA	480
Total			**2,160 (=36** ^**1/2**^ **h)**	**3,723.2**
Working days			**5**	**NA**

NGS: Next-generation sequencing; PGM: Personal Genome Machine PCR: polymerase chain reaction; RQ-PCR: real-time quantitative polymerase chain reaction; SS: Sanger sequencing; HRM: High-resolution melting; NA: Non applicable.

*The duration of the second emulsion PCR required is not included as it is done at the same time as the first sequencing run. **Costs: Cost of consumables and laboratory personal (as cost calculated from the time that the technician/physician is required for the analysis).

## Discussion

In this study we analyzed the spectrum of mutations in 35 melanoma-related genes using a targeted NGS approach on 100 melanoma samples. This in-depth analysis provides important insights into the molecular alterations that contribute to the development and progression of melanoma. In this regard, NGS analysis using commercial pan-cancer panel has been performed in a large cohort of melanoma samples^9^; however as it has been pointed out the majority of genes analyzed in this commercialized cancer panels are not relevant in melanoma pathogenesis, and other recently described genes are not included^10^. Furthermore, in our analysis the deep coverage enabled sensitive discovery of mutations in as low as 0.5% mutant allele frequency, which may be important in FFPE specimens where tumor content may be low and DNA may be degraded. The applicability of FFPE in NGS was previously demonstrated in a study conducted by Chen *et al*.^11^, that sequenced a pair of matched fresh-frozen and FFPE tumor samples and found high concordances between both types of samples. This is relevant as in routine practice FFPE specimens are usually available and fresh-frozen samples are difficult to achieve, especially in melanoma. In addition, as described above and consistent with other reports^12^, sample processing in our hands took approximately three working days, and therefore in comparison to conventional molecular approaches, with NGS the overall time for sample mutation detection is reduced.

Recent WES studies have demonstrated that melanoma has one of the highest rates of somatic mutations among all cancers^13^. In our cohort, we found that the great majority of the analyzed samples (85%) showed at least one somatic mutation among the 35 genes of our customized panel. Moreover, the prevalence of mutations detected in the study among the different melanoma-related genes is in agreement with previous reports. In our primary melanoma series, *BRAF* mutations were the most prevalent (50%) followed by *TERT* promoter mutations (33%). The prevalence of mutations in RAS family was also similar to other studies, as *NRAS* mutations were present in 15% of the samples and *KRAS* in 3%. As previously described, mutations were detected at different frequencies across all melanoma subtypes; *BRAF* and *NRAS* were more frequent in SSM and NM, *KIT* mutations were the most prevalent in ALM, and *TERT* promoter mutations were significantly associated with NM^2, 14^.

Consistent with previous reports, we found mutual exclusivity between somatic mutations in *BRAF* and *NRAS*/*KRAS* in 97% (97/100) of the samples^15^. Interestingly, we also identified concurrent point mutations on the *KRAS* (Q61R and A146V) and *NRAS* (E62Q and Q61R) in two of the samples. To our knowledge these concurrent mutations have not been described before, as previously reported cases show coexisting mutations in codon 12^16^. It has been suggested that these co-mutations may cooperate to activate the MAPK pathway^9^, and therefore the identification could have prognostic and therapeutic implications.

Moreover, we aim to analyze the prevalence of mutations in new recurrently mutated genes in melanoma. Such is the case of *PREX2* (a guanine nucleotide exchange factor and a *PTEN* regulating protein) and *GRIN2A* (a ionotropic glutamate receptor), both recently described in WES studies with mutations distributed along the length of the entire gene, and in frequencies around 14% and 33%, respectively^17, 18^. In our cohort, *PREX2* frequency is consistent with previous reports; however *GRIN2A* prevalence was lower than previously described. In this respect, it is worth mentioning that all variants with a benign *in silico* prediction were excluded. Additionally, we have analyzed the *ERBB4* gene, that was initially found to be somatically mutated in 19% of patients with cutaneous melanoma^19^, although subsequent studies have reported lower prevalences, in coexistence with *BRAF* or *NRAS* mutations^20^. The prevalence of pathogenic variants in our series was 12%, of whom 50% (6/12) were concurrent with *BRAF/NRAS*. Combination strategies for targeted therapies with BRAF inhibitors and ERBB family kinases inhibitors have been pointed out as a promising therapeutic option in the future. In addition, among the newly identified cancer genes are *RAC1* and *PPP6C*. The *RAC1* p.P29S mutation was present in 7% of the samples and as previously described, it was significantly associated with sun-exposed melanomas^21^. This activating mutation may have clinical implications as it has been associated to aggressive melanoma features^22^, and it has been described to regulate PD-L1 expression^23^ and to confer resistance to BRAF inhibitors^24^. Hotspot mutations in *PPP6C* have been found with an overall prevalence of 8%^22, 25^, significantly associated with *NRAS*-mutated melanomas^26^. We have identified an overall prevalence of 6%, with the p.R264C *PPP6C* hotspot in 4% of our samples, mostly in concurrence with *BRAF* mutations rather than *NRAS* mutations. Although several studies have highlighted the relevance of these recently described genes, clinical and therapeutic impact of such genes will need to be determined in future studies.

As recently described by TCGA^3^, our panel provides a genomic classification of melanoma with four different subtypes. It should be noted that we have a higher prevalence in the triple-wt subtype. This could be explained in part due to the lower prevalence in *NF1* gene, as we did not performed WES and therefore our panel did not covered the entire CDS. An alternative explanation of the different prevalence is that our cohort only included primary melanomas.

Considering the recent development of targeted therapies, it is of great importance to identify molecular alterations that contribute to the appearance of resistances in a high percentage of the patients^27^. In this regard, our panel design covers genes that have been related to such mechanisms of resistance, as *MAP2K1*, *MAP2K2*, *MITF*, *PTEN*, *PIK3CA, NF1*, *RAC1* and *HOXD8*. Therefore, application of this melanoma-specific panel could be of interest in the management of metastatic patients, not only for the detection of actionable mutations, but also to identify those patients who may more likely benefit from those treatments.

In summary, the cost-effectiveness (reduced DNA input amount, increase in sensitivity, low turn-around time, and simultaneous analysis of multiple cancer-driving genes) of our new NGS approach based on Ampliseq libraries and Ion PGM sequencing, strongly suggest its implementation in routine diagnostics. In this study we have successfully performed NGS in FFPE melanoma and we have found a wide variety of somatic mutations that may contribute to the pathogenesis of melanoma. Since there are multiple mutations in individual tumors, and each tumor has a specific genetic profile, characterization of the molecular alterations of individual samples seems to be necessary in order to develop a personalized medicine. In addition, application of this panel may also provide further information about the genetic mechanisms of resistance to available therapies, so it would be especially valuable for clinicians in the management of patients with metastatic disease.

## Materials and Methods

### Patients and tumor samples

One hundred melanoma FFPE tumor samples were retrieved from the tissue archives of the Departments of Dermatology and Pathology of the University Hospital La Fe, University General Hospital of Valencia, La Plana Hospital of Villarreal, and Instituto Valenciano de Oncología, Spain. This study was approved by the Institutional Review Board at University Hospital La Fe, University General Hospital of Valencia, La Plana Hospital of Villarreal, and Instituto Valenciano de Oncología, and was carried out in accordance with the approved guidelines. Writen inform consent from all patients were obtained. Main clinicopathological features of the patients are summarized in Table 5.

**Table 5:** Clinicopathological characteristics of the patients included in the study.

Variables	n = 100
Epidemiological features	
Age at diagnosis [median, (range)]	65.5 (21–90)
Age at diagnosis (%)	
<40	6
40–65	44
>65	50
Sex (%)	
Male	53
Female	47
Clinical melanoma features	
Anatomic site (%)	
Head/neck	16
Upper extremities	16
Trunk	42
Lower extremities	18
Acral	8
Sun related site (%)	
Non exposed	21
Occasionally exposed	63
Usually exposed	16
Pathological melanoma features	
Histological subtype (%)	
LMM	10
SSM	68
NM	14
AML	8
Breslow thickness (mean (±SD))	1.9 ± 2.1
Breslow thickness (%)	
<1 mm	47
1–2 mm	21
2–4 mm	19
>4 mm	13
Ulceration (%)	
No	74
Yes	26
Regression (%)	
No	52
<50%	41
>50%	7
Mitoses/mm2 (%)	
<1	48
>1	52
Growth rate (%)	
SGM	76
FGM	24
Tumor stage (%)	
Localized (I–II)	75
Locoregional and metastatic disease (III–IV)	25
Months follow-up [median, (range)]	32 (6–121)
Clinical outcome (%)	
Stable disease	81
Relapse (locoregional vs distant metastasis)	8
Exitus	11

### DNA preparation

Genomic DNA was isolated from two 10-μm thick FFPE sections using Deparaffinization Solution and the GeneRead DNA FFPE Kit (Qiagen, Hilden, Germany) according to manufacturer’s protocol. This isolation kit contains Uracil-DNA-Glycosylase (UNG) treatment that prevents formalin-fixation induced artifacts, which may lead to false-positive mutation callings^28^. DNA concentration was quantified by Qubit dsDNA HS Assay Kit (ThermoFisher Scientific). Genomic DNA with at least 10 ng/μL was subjected to library preparation.

### Integrative molecular analysis by Next-generation sequencing

#### Melanoma-specific panel design

A custom panel was designed using Ion AmpliSeq Designer platform (www.ampliseq.com) to cover the coding regions of 35 melanoma-related genes (*NRAS, ERBB4, HOXD8, ALK, MITF, BPA1, PIK3CA, KIT, TERT, EPHA7, STK19, BRAF, MET, GRM3, RAC1, STK31, TRRRAP, PREX2, CDKN2A, GNAQ, TAF1L, PPP6C, PTEN, HRAS, KRAS, CDK4, MAP2K1, MC1R, GRIN2A, ADAMST18, NF1, GNA11, MAP2K2, STK11, IRS4*). The selection of the genes to be included in this panel was based on the relatively high frequency of mutations over other whole-exome sequencing (WES) studies, as well as their possible diagnostic, prognostic and therapeutic implication. In five genes, sequencing of the complete coding sequence (CDS) was performed, whereas in the other 30 genes specific targeted exonic regions were studied, focusing on areas where hotspot mutations are known to occur. The panel covered 23,702 base pairs (bp) with an average coverage of 97.8%. A total of 515 amplicons were designed with an amplicon range of 125–175 bp. All panel details are summarized in Table S2.

#### Ion Torrent Library preparation

Library preparation was performed using the Ion Ampliseq Library kits 2.0 (Thermo Fisher Scientific). Multiplex PCR amplification of 10 ng of DNA was performed using the custom *Ion AmpliSeq Primer Pool* and the *Ion AmpliSeq Hifi Mix*. (Thermo Fisher Scientific) according to manufacturer’s protocol. Primer sequences were partially digested using *FuPa Reagent*, and *Ion Torrent adapters* and *Ion Xpress Barcodes* were ligated with DNA ligase. Following adapter’s ligation, amplicons were purified with *Agencourt® AMPure® XP* (Beckam Coulter), and subsequently quantification of the amplified library was performed using *Ion Library Equalizer Kit* (ThermoFisher Scientific) according to manufacturer’s protocol.

#### Emulsion PCR and DNA sequencing

The library pool was clonally amplified in an emulsion PCR reaction using Ion Sphere Particles (ISPs) on the One Touch 2 Instrument, and subsequently template-positive ISPs were enriched on the Ion One Touch ES (Thermo Fisher Scientific) as described by the manufacturer. Enriched template-positive ISPs were subjected to sequencing on the Ion Torrent PGM on a 318v2 Chip (four samples per chip) (Thermo Fisher Scientific).

#### Sensitivity assay

Sensitivity was assessed by sequencing serially diluted DNA isolated from two cell lines: DNA isolated from HT-29 (ACC-299), a human colorectal adenocarcinoma cell line with a known heterozygous mutation in *BRAF* (p.V600E) and a polymorphism in *MC1R* (p.R160W), was diluted into DNA obtained from another colorectal adenocarcinoma cell line Caco-2 (ACC-169), known to be wild-type for the mentioned variations, in ratios of 1:1, 1:3, 1:24, 1:49, 1:99, 1:999 resulting in 25%, 12.5%, 2%, 1%, 0.5% and 0.05% dilutions of the mutated allele, respectively. To asses reproducibility sequencing runs of the diluted DNA were performed in two independent experiments.

#### Variant Calling and experimental validation

Data from sequencing runs were transferred to the Torrent Server, and Ion Torrent Suite Software was used to generate initial variant calling. Filtered variants were annotated using Ion Reporter software. Exclusion of non-exonic variants and synonymous mutations was carried out. Subsequently, several steps were used in order to filter out variants with low read numbers: a minimum depth of total coverage ≥500 reads, an each variant coverage of ≥20 reads, and P-value < 0.01. Moreover, mutations were visually examined using Integrative Genomics Viewer (IGV) software. In addition, detected missense mutations in *BRAF, NRAS, KRAS* and *KIT* were subsequently confirmed by different methods. *BRAF* mutations were validated by High Resolution Melting (HRM) as previously described^29^, and Sanger’s sequencing*; NRAS* and *KRAS* mutations were confirmed by RQ-PCR using AmoyDx® KRAS/NRAS Mutations Detection Kit, according to manufacturer’s instructions; and *KIT* mutations were validated by direct sequencing.

#### Prediction tools analysis

Databases such as COSMIC, TCGA and dbSNP were used to assess recurrent known mutations and to exclude reported germline polymorphisms. Furthermore, in all the variants of unknown significance we aimed to identify those ones likely to impact protein function using five prediction tools such as Provean, SIFT (Sorting Intolerant From Tolerant), PolyPhen-2 (Polymorphism Phenotyping v2), SNPS&GO, and Condel, which use algorithms that predict the effect of amino acid substitution on the protein structure and function. We excluded the variants predicted as “benign” by at least three of the five applied prediction tools.

### Detection of *TERT* promoter mutations by direct sequencing

Mutational status of the *TERT* promoter region (from position −27 to −286 from ATG start site) was determined by PCR and Sanger sequencing. A 260 bp region was amplified using a pair of primers previously described^30^. The PCR was performed in a conventional thermal cycler using the following cycling conditions: initial heating at 95 °C for 5 min followed by 40 cycles of 45 s denaturation at 95 °C, 45 s annealing at 59 °C, 36 s extension at 72 °C, and, finally, 72 °C for 10 min. PCR product was subsequently sequenced on the ABIprism 3130 (Applied Biosystems).

### Statistical analysis

Quantitative variables were summarized by their mean and standard deviation, and categorical variables by relative and absolute frequencies. The relationship between mutations and clinicopathological features was evaluated using logistic regression analysis with estimation of OR and 95% CI. P values < 0.05 were considered statistically significant. Computations were performed using the SPSSv21.statistical package (Chicago, IL).

## Electronic supplementary material

Supplementary information II

Supplementary information I
