# Supplementary material for: Towards Personalized Medicine in Melanoma: Implementation of a Clinical Next-Generation Sequencing Panel
Source: Sci Rep. 2017 Mar 29;7:495. doi: 10.1038/s41598-017-00606-w (PMC5428782; doi:10.1038/s41598-017-00606-w)
Supplement: Supplementary file 1 — Supplementary information II [file 41598_2017_606_MOESM1_ESM.doc]

**TITLE**

Towards Personalized Medicine in Melanoma: Implementation of a Clinical Next-Generation Sequencing Panel.

**AUTHORS AND AFFILIATION**

Blanca de Unamuno Bustos^1#^, MD, Rosa Murria Estal^2#^, PhD, Gema Pérez Simó^2^, BSc, Inmaculada de Juan Jimenez^2^, PhD, Begoña Escutia Muñoz^1^, MD, PhD, Mercedes Rodríguez Serna^1^, MD, PhD, Victor Alegre de Miquel^3^, MD, PhD, Margarita Llavador Ros^4^, MD, Rosa Ballester Sánchez^5^, MD, Eduardo Nagore Enguídanos^6^, MD, PhD, Sarai Palanca Suela^2*†^ PhD, Rafael Botella Estrada^1*^, MD, PhD.

# co-authors (contributed equally to the manuscript)

* co-senior investigators

†Correspondence should be addressed to [palanca_sar@gva.es](mailto:palanca_sar@gva.es) and saraipalanca@hotmail.com

^1^Department of Dermatology, Hospital Universitari i Politecnic La Fe, Valencia (Spain).

^2^Molecular Biology Laboratory, Service of Clinical Analysis, Hospital Universitari i Politecnic La Fe, Valencia (Spain).

^3^Department of Dermatology, Hospital General Universitario de Valencia, Valencia (Spain).

^4^Department of Pathology, Hospital Universitari i Politecnic La Fe, Valencia (Spain).

^5^Department of Dermatology, La Plana Hospital, Villarreal, Castellón (Spain).

^6^Department of Dermatology, Department of Dermatology, Instituto Valenciano de Oncología, Valencia (Spain).

**SUPPLEMENTARY INFORMATION**

**Supplementary table 1**

(Please see accompanying Excel file “Table S1 – Pathogenic variants”)

**Supplementary table 2**

(Please see accompanying Excel file “Table S2 – Custom panel design”)


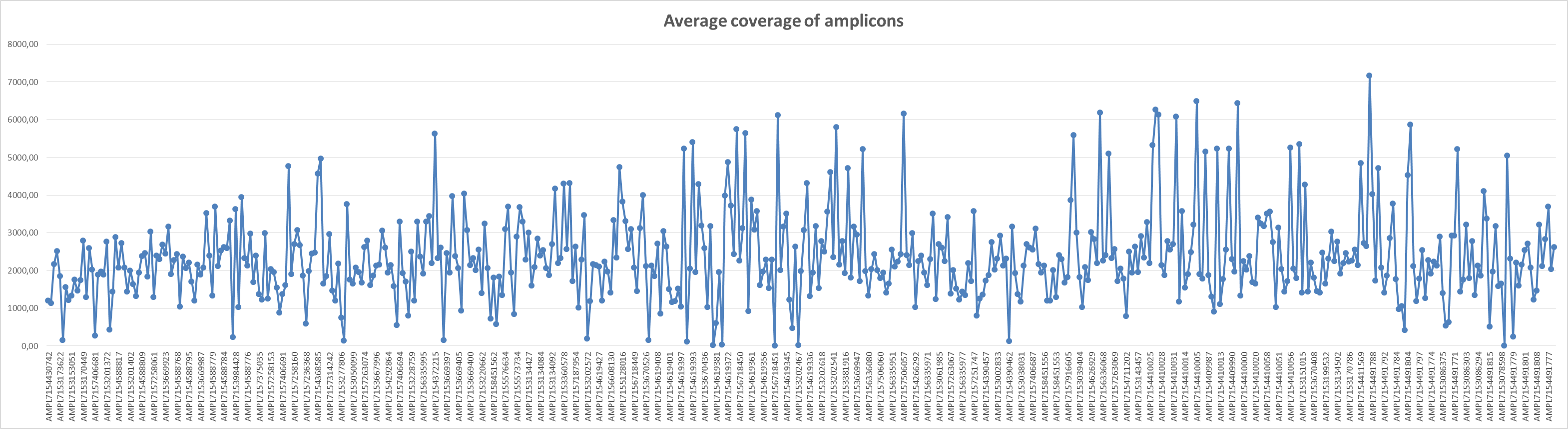
**Figure S1**

**Figure S1:** Read coverage across 515 amplicons. Average coverage of 2,575.5 reads per amplicon.

**Figure S2**


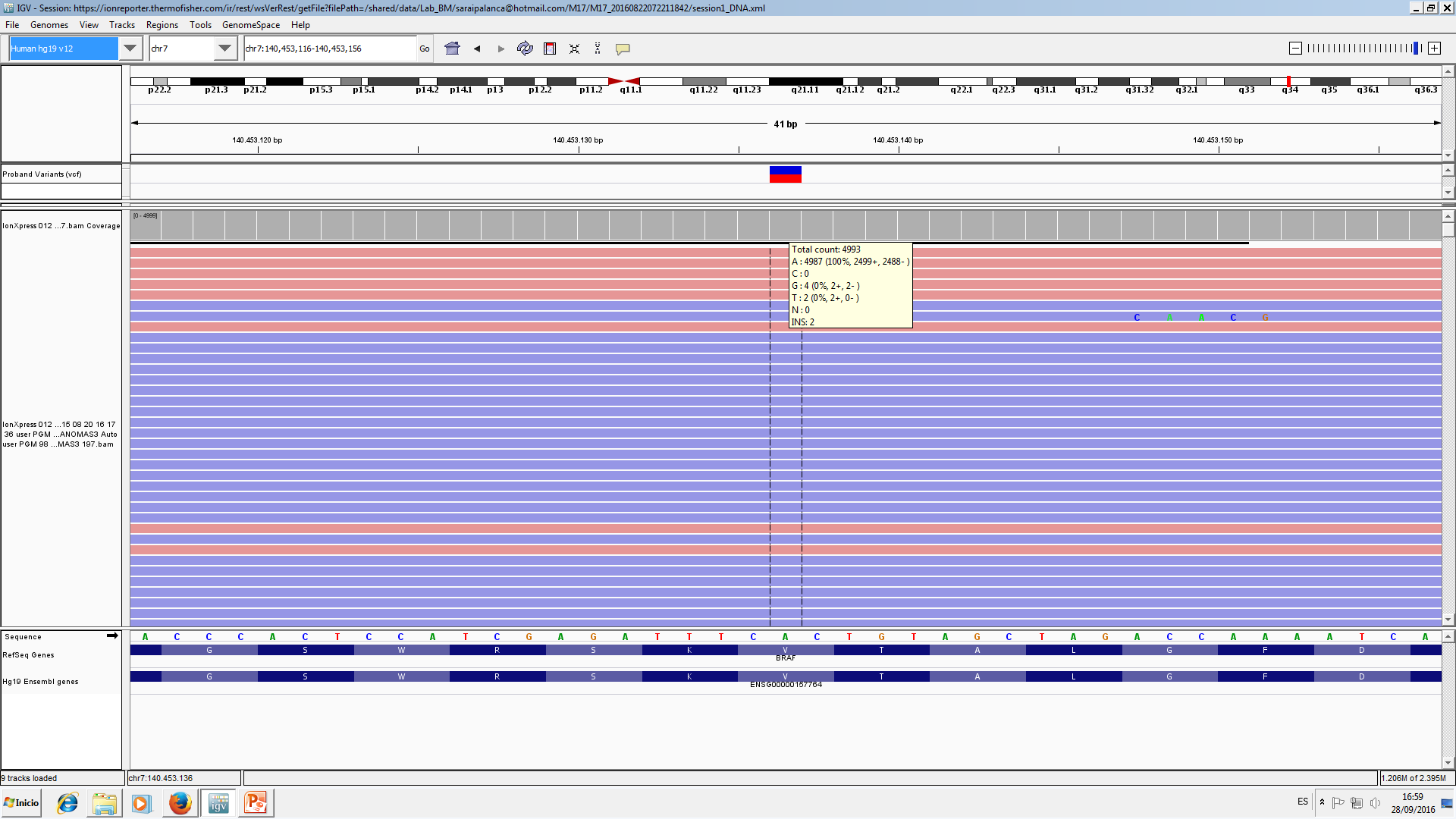

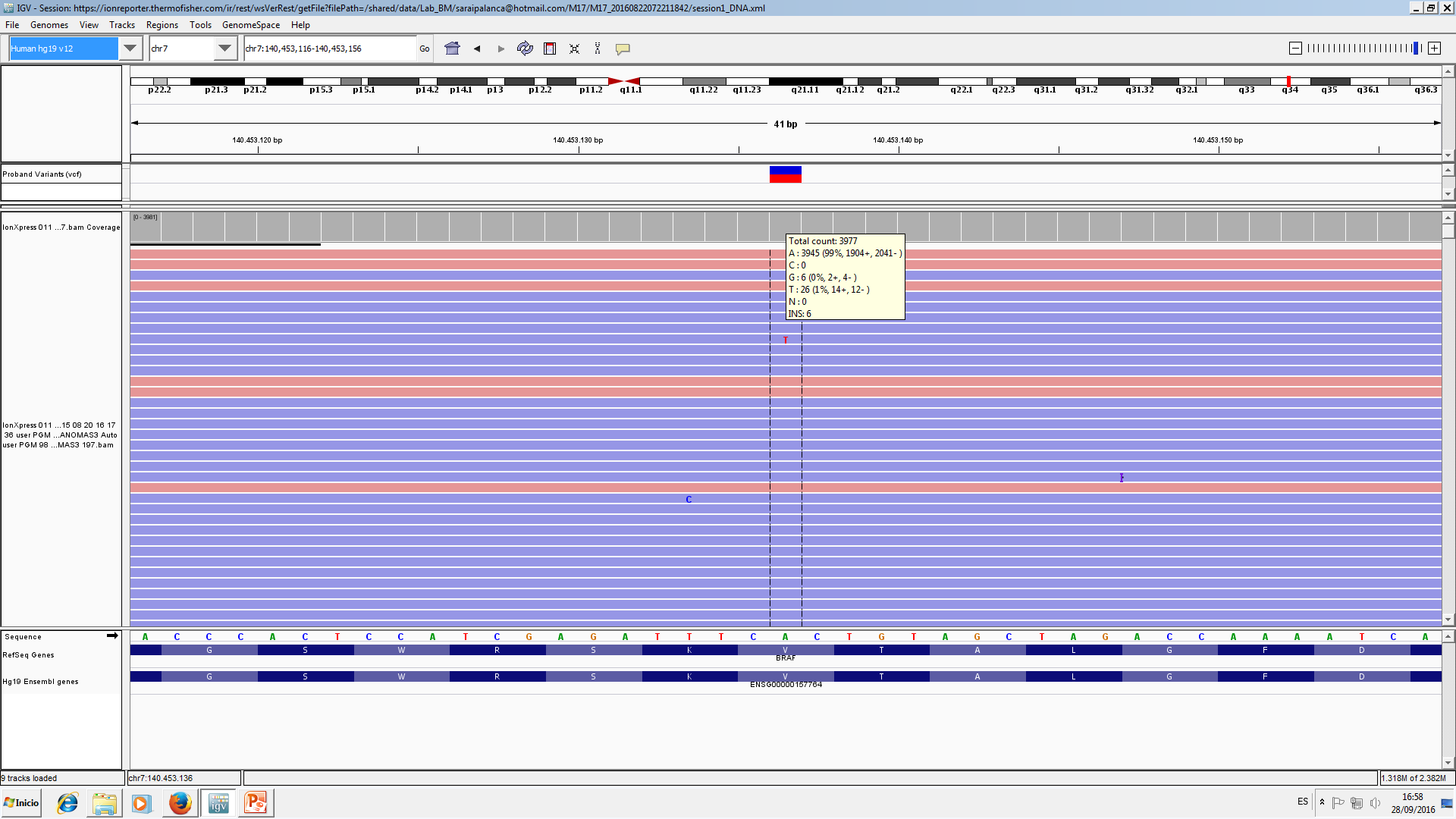

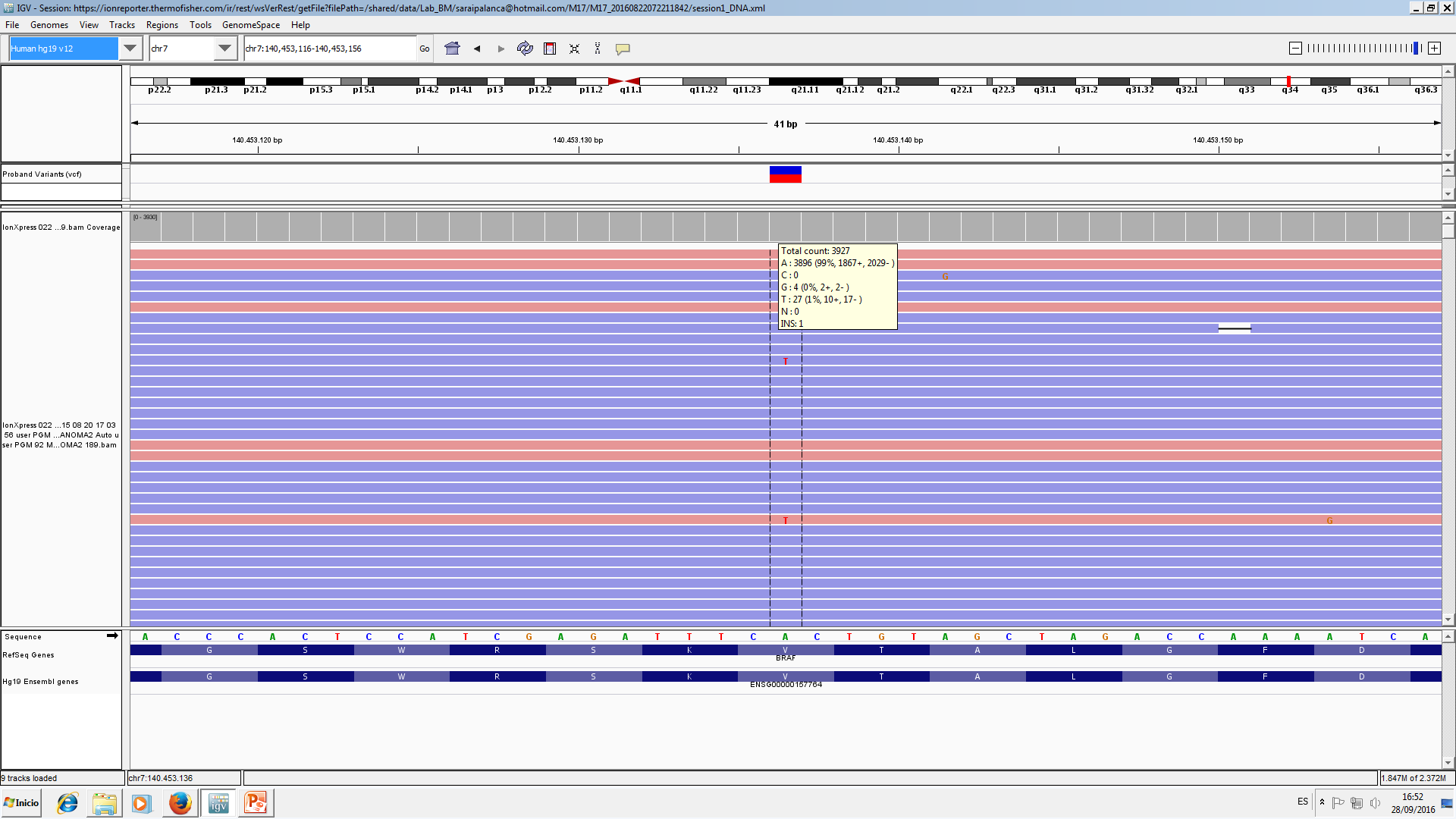

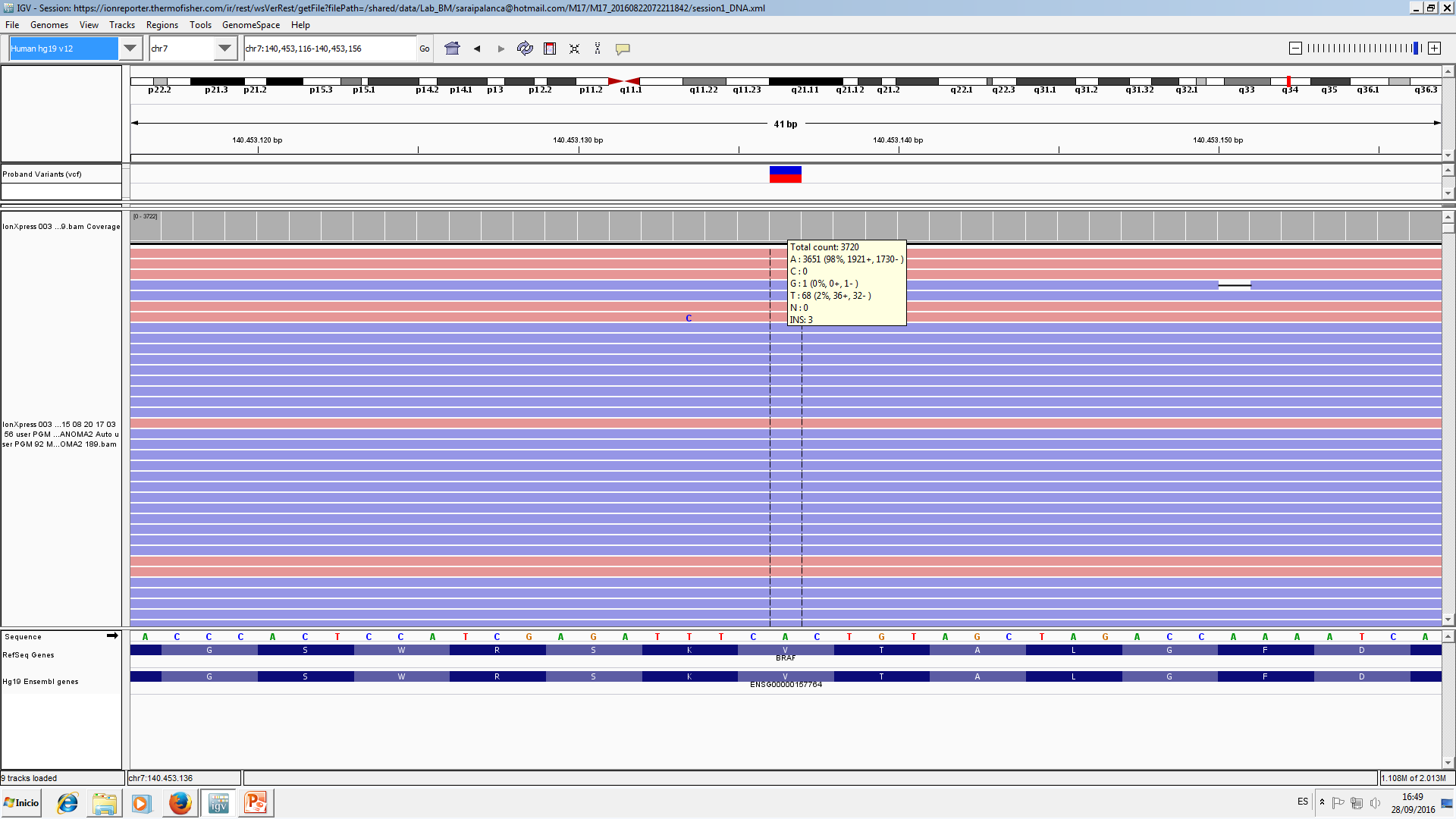

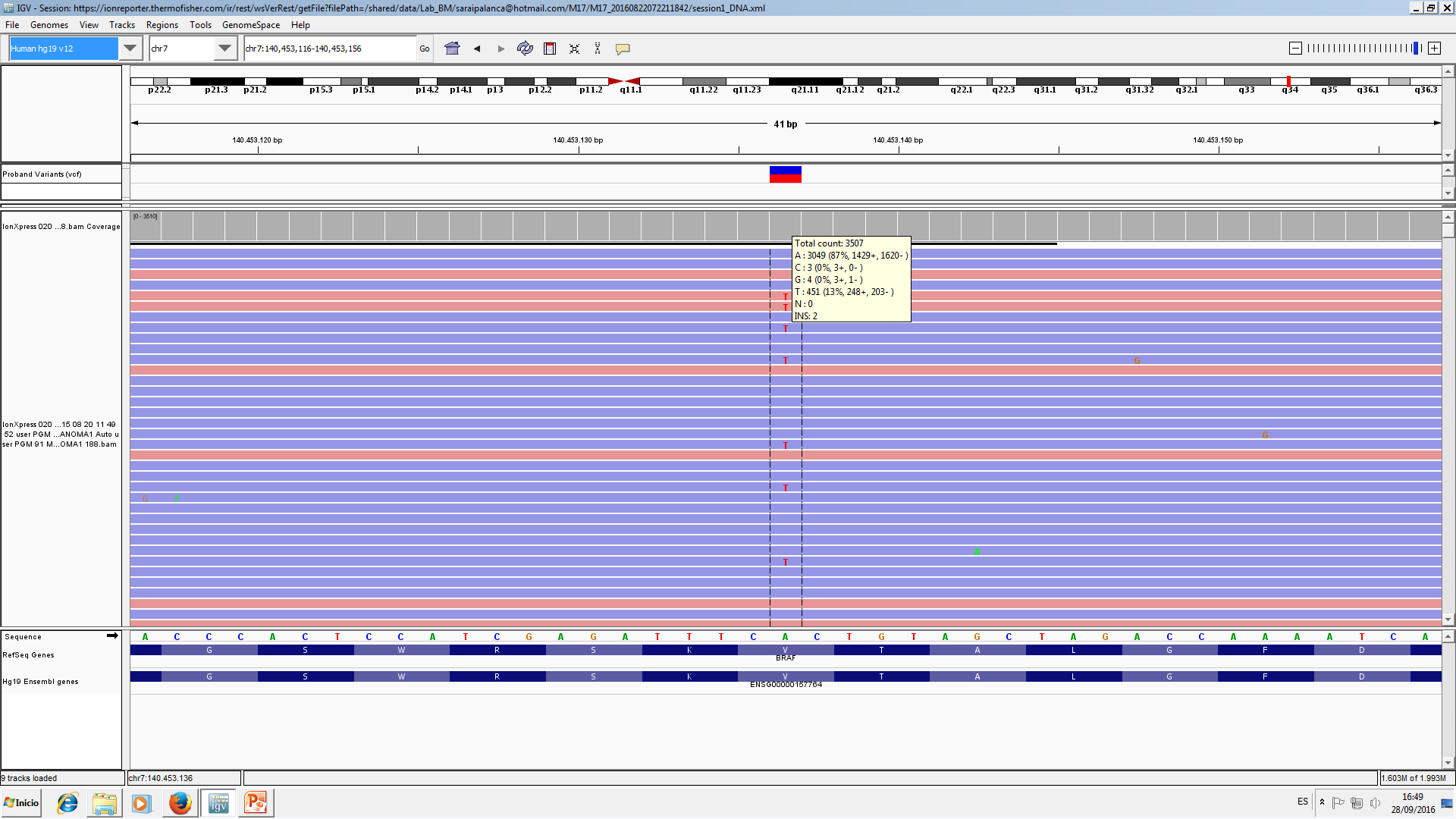

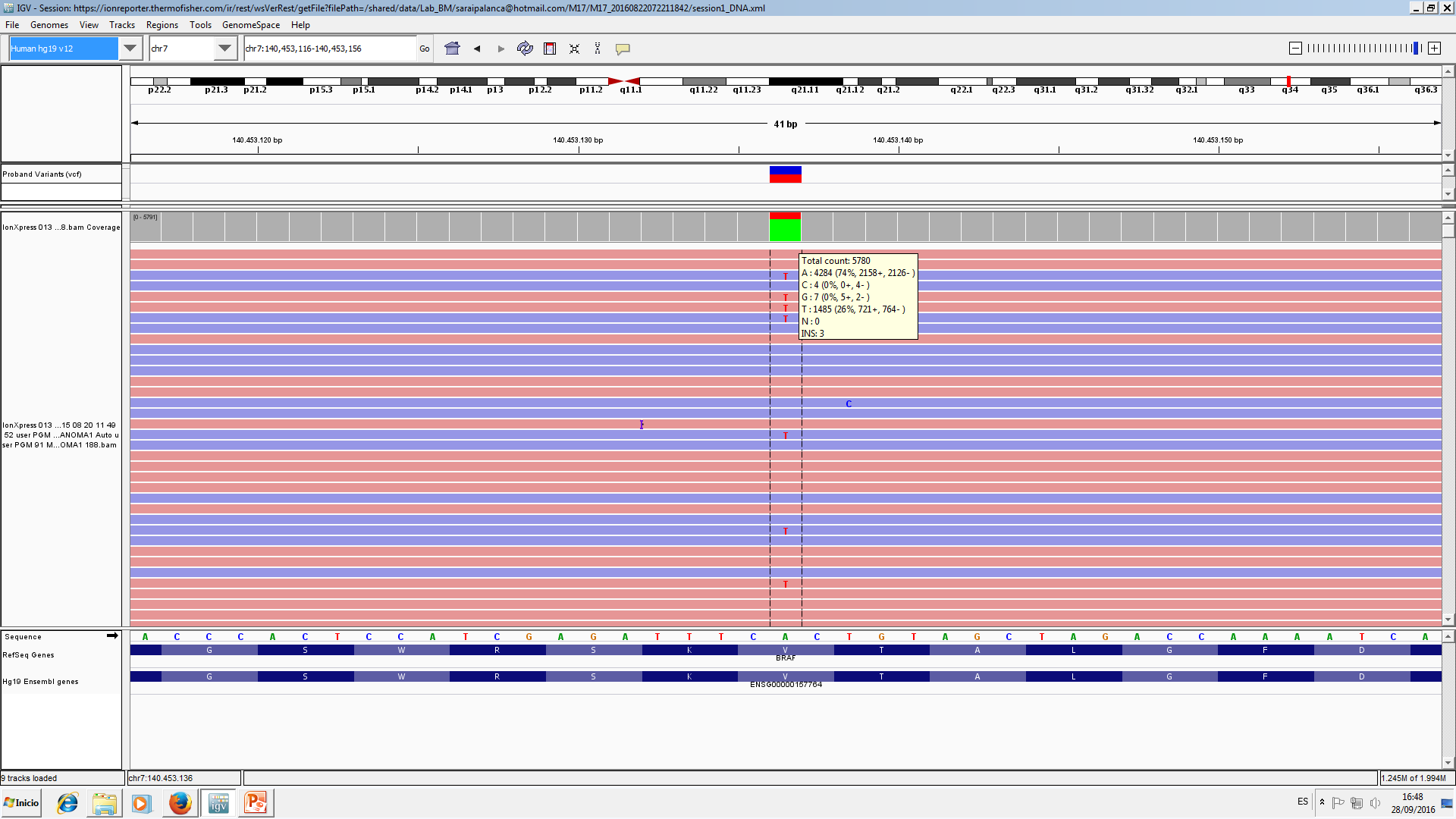


**25%**

**2%**

**12.5%**

**1%**

**0.5%**

**0.05%**

**Figure S2:** IGV visualization of the *BRAF* mutation p.V600E (c.1799A>T) in serially diluted DNA (1:1, 1:3, 1:24, 1:49, 1:99, 1:999 resulting in 25%, 12.5%, 2%, 1%, 0.5% and 0.05% dilutions of the mutated allele) isolated from two cell lines (HT-29 and Caco-2). The mutation was detected in the forward (red in IGV) and reverse (blue in IGV) strands.


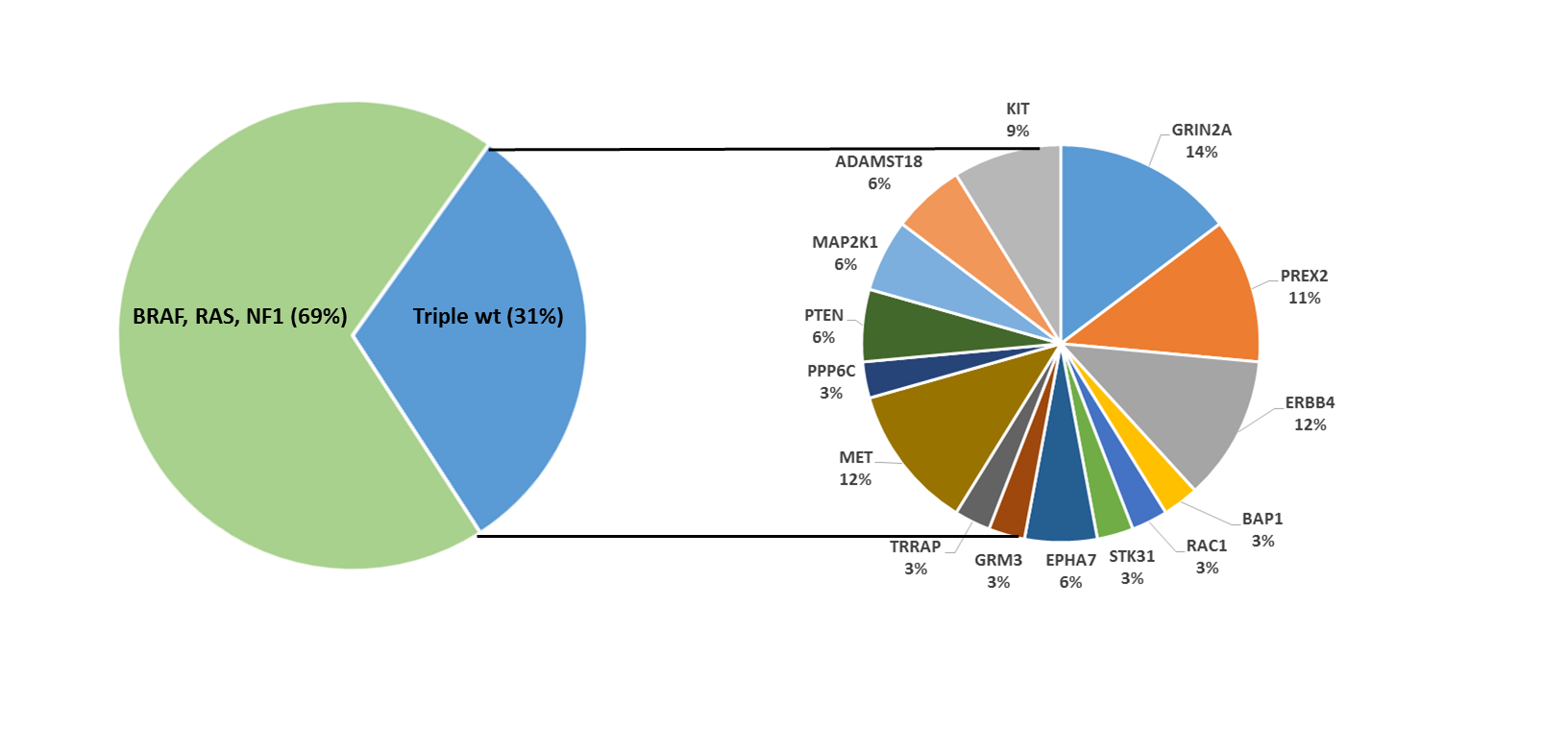
**Figure S3:**

**Figure S3**: Classification in four genomic subtypes [BRAF, RAS, NF1 (69%; 69/100) and triple-wt (31 %; 31/100)]. Prevalence of mutations in the triple wt subtype.

**Figure S4**

**
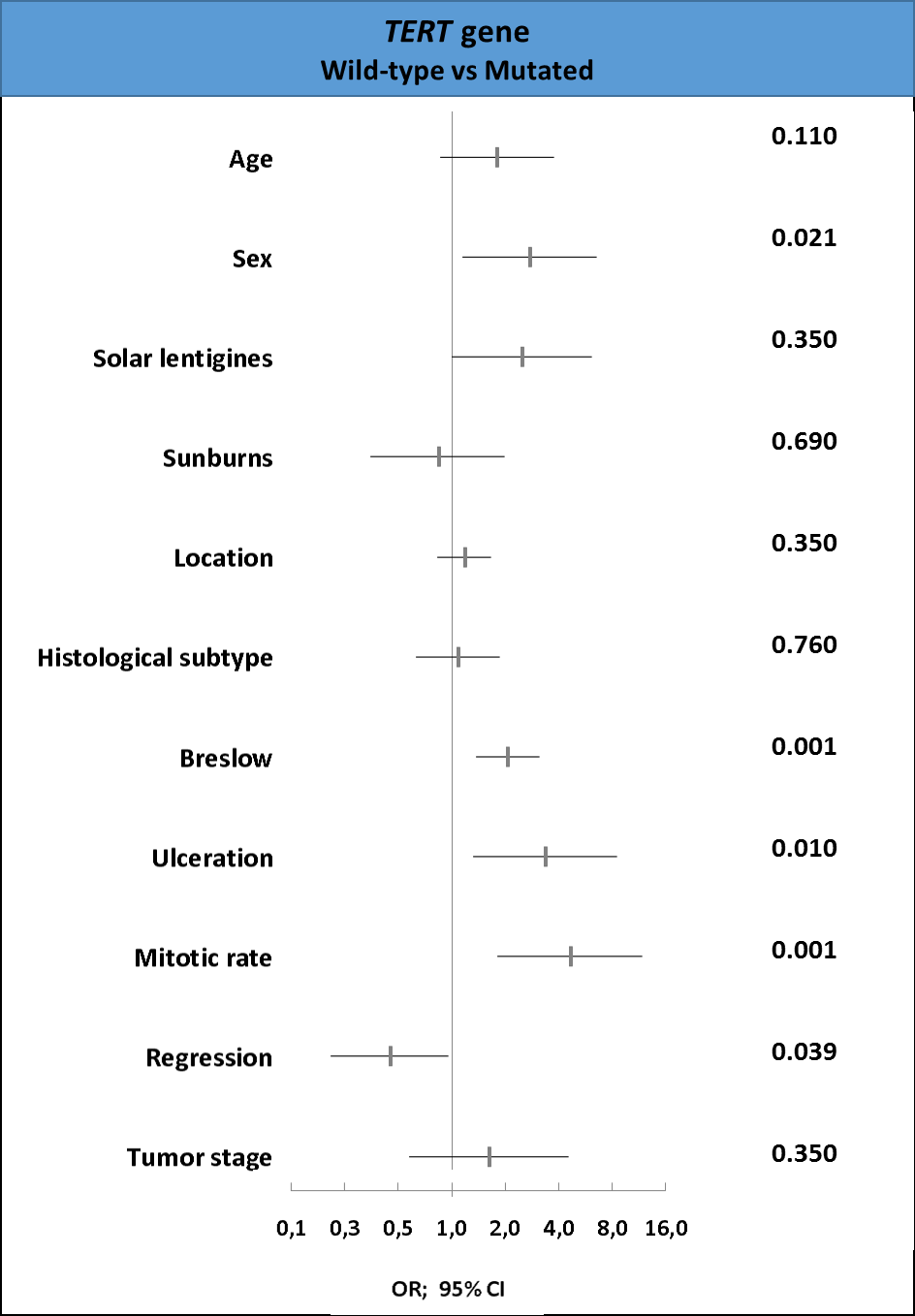
**

Figure S4: Associations of *TERT* promoter mutation status and clinicopathological features**.** P values were derived from logistic regression and considered statistically significant if <0.05.
